# Examination of diverse iron-chelating agents for the protection of differentiated PC12 cells against oxidative injury induced by 6-hydroxydopamine and dopamine

**DOI:** 10.1038/s41598-022-13554-x

**Published:** 2022-06-13

**Authors:** Pavlína Hašková, Lenka Applová, Hana Jansová, Pavel Homola, Katherine J. Franz, Kateřina Vávrová, Jaroslav Roh, Tomáš Šimůnek

**Affiliations:** 1 Faculty of Pharmacy, Charles University, Akademika Heyrovského 1203, 500 05 Hradec Králové, Czech Republic; 2 Department of Chemistry, Duke University, Durham, NC USA

**Keywords:** Iron, Parkinson's disease, Pharmacodynamics, Small molecules, Cell death, Drug screening, Medicinal chemistry, Toxicology

## Abstract

Labile redox-active iron ions have been implicated in various neurodegenerative disorders, including the Parkinson's disease (PD). Iron chelation has been successfully used in clinical practice to manage iron overload in diseases such as thalassemia major; however, the use of conventional iron chelators in pathological states without systemic iron overload remains at the preclinical investigative level and is complicated by the risk of adverse outcomes due to systemic iron depletion. In this study, we examined three clinically-used chelators, namely, desferrioxamine, deferiprone and deferasirox and compared them with experimental agent salicylaldehyde isonicotinoyl hydrazone (SIH) and its boronate-masked prochelator BSIH for protection of differentiated PC12 cells against the toxicity of catecholamines 6-hydroxydopamine and dopamine and their oxidation products. All the assayed chelating agents were able to significantly reduce the catecholamine toxicity in a dose-dependent manner. Whereas hydrophilic chelator desferrioxamine exerted protection only at high and clinically unachievable concentrations, deferiprone and deferasirox significantly reduced the catecholamine neurotoxicity at concentrations that are within their plasma levels following standard dosage. SIH was the most effective iron chelator to protect the cells with the lowest own toxicity of all the assayed conventional chelators. This favorable feature was even more pronounced in prochelator BSIH that does not chelate iron unless its protective group is cleaved in disease-specific oxidative stress conditions. Hence, this study demonstrated that while iron chelation may have general neuroprotective potential against catecholamine auto-oxidation and toxicity, SIH and BSIH represent promising lead molecules and warrant further studies in more complex animal models.

## Introduction

Iron (Fe) is the most abundant transition metal in the human body with a fundamental role in various physiological processes. It serves as an electron donor and acceptor due to its easy cycling between its ferrous (Fe^2+^) and ferric (Fe^3+^) oxidation states. Iron participates in a wide variety of biochemical processes, including DNA synthesis, mitochondrial electron transport and oxidative phosphorylation, and its proper homeostasis is therefore vital for normal function of the central nervous system^1^. Fe is distributed heterogeneously in different brain regions. It is involved in neurotransmitter metabolism, including dopamine (DA) synthesis via stimulation of tyrosine hydroxylase activity^2^.

On the other hand, labile and redox-active Fe ions can catalyze the Fenton reaction yielding hydroxyl radicals—the most reactive and toxic form of reactive oxygen species (ROS)^1,3^. Increased Fe levels, primarily in substantia nigra zona compacta in the ventral midbrain, have been associated with neurodegeneration in Parkinson’s disease (PD), a severe progressive neurological disorder that primarily affects movement control^2^.

DA serves as a neurotransmitter in the neuronal communication between the substantia nigra and the basal ganglia. In PD, the substantia nigra neurons progressively degenerate and amount of DA available for neurotransmission in the corpus striatum is lowered. Furthermore, DA can undergo oxidation or auto-oxidation to form semiquinones and quinones, i.e. processes that contribute to the reduction of Fe ions and formation of hydroxyl radicals^4^. The formed ROS cause damage to essential macromolecules, which can lead to a critical failure of biological functions, protein modification, misfolding, and ultimately neuronal death^5^.

For decades, Fe chelators have been successfully used in clinical practice in Fe-overload syndromes, such as those caused by repeated blood transfusions in patients with β-thalassemia major^6^. Recently, there has been increasing evidence of the positive effect of Fe chelators in the treatment of diseases characterized by oxidative stress associated with a local release of Fe, including post-ischemic reperfusion injury of myocardium, intoxications by xenobiotics, inflammatory disorders, atherosclerosis or cancer^6–8^. Furthermore, even clinical trials have reported the potential of various Fe chelators to slow progression or improvement in neurodegeneration^9,10^. Three Fe chelators currently approved for clinical use, namely the hexadendate deferoxamine (DFO), the bidentate deferiprone (L1), and the tridentate chelator deferasirox (ICL670A), were previously described as protective, in vitro as well as in vivo, against dopaminergic neurodegeneration induced by 6-hydroxydopamine (6-OHDA)^11,12^, 1-methyl-4-phenyl-1,2,3,6-tetrahydropyridine (MPTP)^13,14^ as well as against model oxidative injury induced by hydrogen peroxide and *tert*-butylhydroperoxide^14–16^.

All the three chelators in clinical practice have been approved for the removal of excess iron from the body. In the pathological states with no iron overload, other agent(s) may be more advantageous and useful. Furthermore, the approved drugs suffer from disadvantages and/or adverse reactions and search for new effective iron chelators with low toxicity is still an important objective^17,18^.

In the current study, we firstly compared the neuroprotective potential of these three established chelating agents with experimental tridentate lipophilic and orally active Fe chelator salicylaldehyde isonicotinoyl hydrazone (SIH) using differentiated PC12 cells challenged by 6-OHDA^19,20^. SIH has previously shown a promising cytoprotective effect on H9c2 cells, rat isolated cardiomyocytes and retinal pigment epithelial cells against damage caused by various prooxidants such as hydrogen peroxide, *tert*-butylhydroperoxide, paraquat as well as catecholamines^21–24^. SIH also protected cells against ionizing radiation^25^ and cardiotoxicity of anthracycline antibiotics in vitro as well as in vivo^26,27^.

In the second series of experiments, we studied SIH in more detail, together with its prochelator BSIH, which contains a boronic ester in place of a phenolic hydroxyl, which is a key metal-binding site for SIH. The concept of Fe prochelation seems to be a beneficial strategy especially for pathological states that are not associated with systemic Fe overload. BSIH does not bind Fe ions until the protective boronyl mask is removed by reaction with ROS under conditions specific to diseases associated with oxidative stress^28^. BSIH is nontoxic and more stable compared to parent chelator SIH^29,30^.

To assess and compare the protective potential as well as own toxicities of these various types of chelating agents, we used PC12 rat pheochromocytoma cells that underwent neuronal differentiation in response to nerve growth factor^31^. These differentiated PC12 cells have been widely applied model for in vitro studies related to PD as they synthesize, store and can be stimulated to release DA. Furthermore, they resemble sympathetic neurons’ phenotype as they extend axons, are electrically excitable and respond to neurotransmitters^32,33^. Furthermore, the differentiated PC12 cells are known to respond well to model toxins used also in vivo such as 6-OHDA and MPTP^12,20^. 6-OHDA is a specific catecholaminergic neurotoxin structurally analogous to both DA and noradrenaline. Under physiological conditions, 6-OHDA undergoes rapid and nonenzymatic auto-oxidation^34^ to generate several toxic species including quinones, superoxide radicals, hydrogen peroxide and hydroxyl radical^35^.

Apart from the parent catecholamines DA and 6-OHDA, this study examined the toxicities of their auto-oxidation products and protective effects of Fe (pro-)chelators on catecholamine autoxidation and intracellular ROS formation.

## Methods

### Chemicals

**Figure 1:**
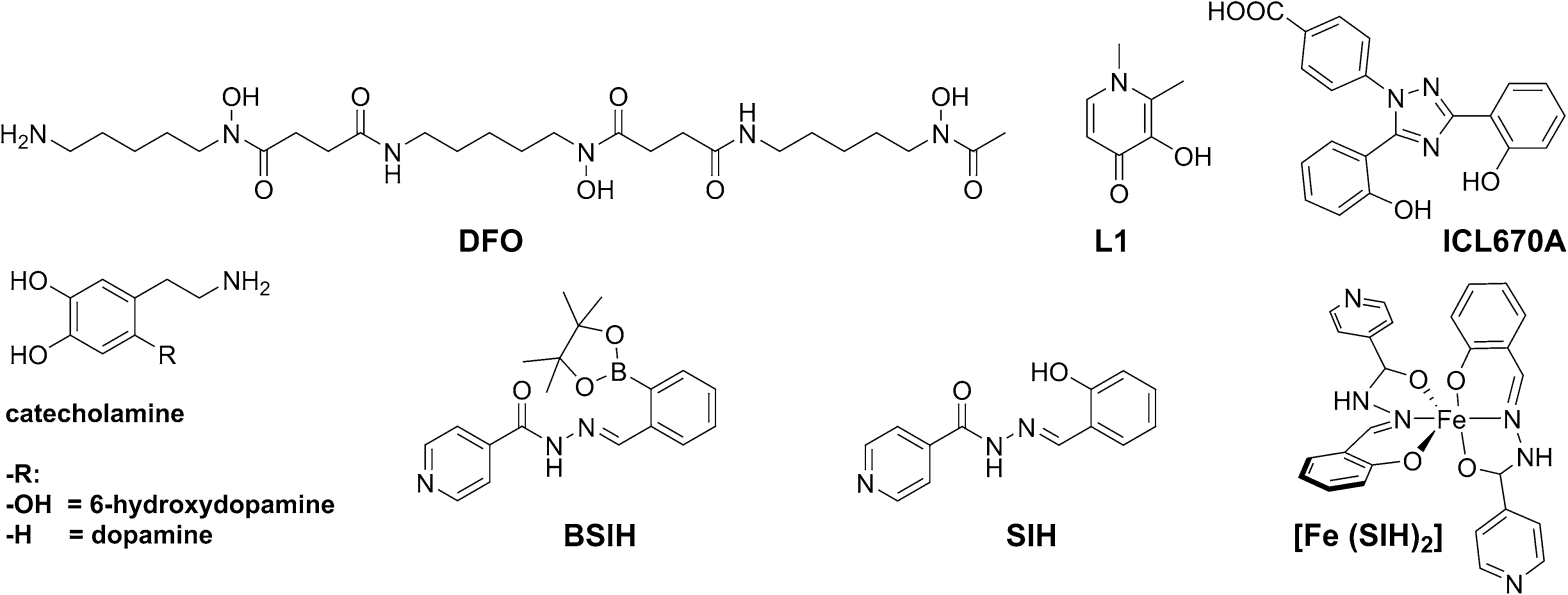
Structures of investigated compounds. Fe chelators used in clinical practice: desferrioxamine (DFO), deferiprone (L1) and deferasirox (ICL670A); catecholamines: 5-(2-aminoethyl)benzene-1,2,4-triol (6-hydroxydopamine) and 4-(2-aminoethyl)benzene-1,2-diol (dopamine); Fe prochelator: (*E*)-N′-(2-(4,4,5,5-tetramethyl-[1,3,2]dioxaborolan-2-yl)-benzylidene) isonicotinohydrazide (BSIH); Fe chelator: (*E*)-N′-(2-hydroxybenzylidene) isonicotinohydrazide (SIH) and its complex with Fe [Fe(SIH)_2_].

The catecholamines 6-hydroxydopamine (5-(2-aminoethyl)benzene-1,2,4-triol; 6-OHDA, Fig. 1) and dopamine (4-(2-aminoethyl)benzene-1,2-diol; DA, Fig. 1), as well as other chemicals (e.g*.* constituents of various buffers) were purchased from Sigma-Aldrich/Merck (Germany) or Penta (Czech Republic) and were of the highest available pharmaceutical or analytical grade. Fe chelators DFO and ICL670A were purified from commercial pharmaceutical preparations of Novartis (Switzerland), L1 substance was a gift of ApoPharma (Canada). The Fe chelator (*E*)-*N*′-(2-hydroxybenzylidene) isonicotinohydrazide (SIH, Fig. 1) and the prochelator (*E*)-*N*′-(2-(4,4,5,5-tetramethyl-[1,3,2]dioxaborolan-2-yl)-benzylidene) isonicotinohydrazide (BSIH, Fig. 1) were synthesized as described previously^36,37^ and their identity and purity were confirmed with elemental analysis, ^1^H and ^13^C NMR, and IR spectroscopy. The chemicals and solutions used for cellular cultivation (e.g*.,* cell culture media, sera) were purchased from Sigma-Aldrich/Merck (Germany) or Lonza Group (Switzerland).

### Cell culture

The PC12 cell line derived from rat phaeochromocytoma tissue^31^ was obtained from the American Type Culture Collection (ATCC, VA, USA). Cells were maintained in culture as naïve cells in RPMI-1640 medium (Sigma-Aldrich/Merck, Germany) supplemented with 10% heat-inactivated horse serum (Sigma-Aldrich/Merck, Germany), 5% heat-inactivated fetal bovine serum (Sigma-Aldrich/Merck, Germany), and 1% penicillin/streptomycin solution (Lonza Group, Switzerland) in 75 cm^2^ tissue culture flasks (TPP, Switzerland) at 37 °C in a humidified atmosphere of 5% CO_2_ in the air. Cells were subcultured usually once a week when they reached approximately 60% confluence.

For experiments with differentiated cells, naïve cells were seeded into appropriate microplates (TPP, Switzerland) at given cellular density in medium for differentiation, consisting of RPMI-1640 medium supplemented with 1% heat-inactivated fetal bovine serum, 1% penicillin/streptomycin solution, and 50 ng/ml nerve growth factor (Sigma-Aldrich/Merck, Germany). The half volume of medium was changed for fresh differentiation medium every 2^nd^-3^rd^ day, and cells were taken into experiments at the 7th–8th day of differentiation.

To dissolve the lipophilic compounds (ICL-670A, SIH, BSIH), dimethyl sulfoxide (DMSO; Sigma-Aldrich/Merck, Germany) was present in a final concentration of 0.1% in all experimental groups. At this concentration, DMSO had no effect on cellular viability.

### Experimental protocols

**Figure 2:**
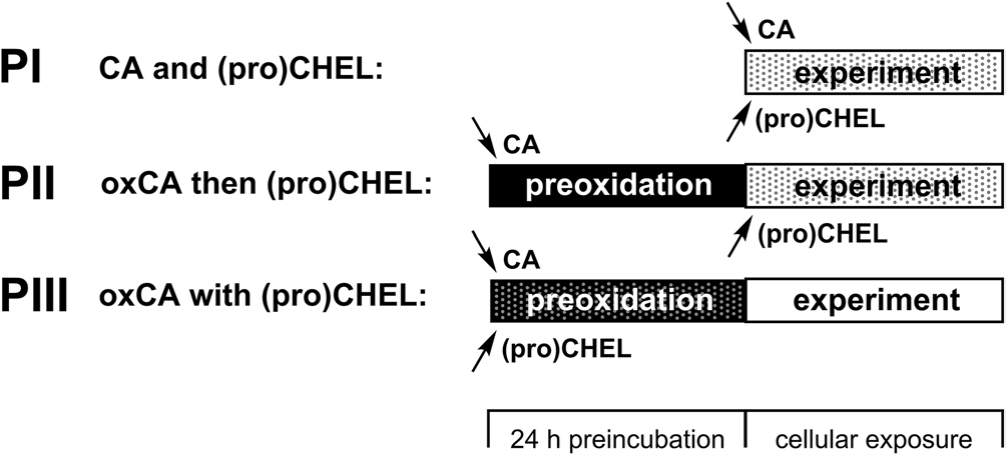
Overview of the cytotoxicity protocols used in this study. Work solutions of catecholamine (CA—6-hydroxydopamine or dopamine) were either freshly-prepared before the cellular exposure (protocol **PI**) or 24 h-preincubated (i.e., left spontaneously oxidize; oxCA) at 37 °C (protocols **PII** and **PIII**). The Fe chelator or prochelator under investigation [(pro)CHEL] was added to the solution with catecholamine either at the start of the cellular exposure (protocols **PI**, **PII**) or at the beginning of 24 h preincubation of catecholamine (protocol **PIII**).

Catecholamines are able to undergo spontaneous oxidation to various products that have been implicated in their toxicity. In this study, three different protocols were used to assess the potential protective properties of compounds under investigation (Fig. 2). Work solutions of catecholamines (“CA”; DA or 6-OHDA) used in experiments were either freshly prepared before the cellular exposure (protocol **P I**) or 24 h-preoxidized (i.e., left to spontaneously oxidize in an incubator for 24 h at 37 °C; “oxCA”; protocols **P II** and **P III**). The Fe chelator or prochelator was added into the solution with catecholamine either at the start of the cellular exposure (protocols **P I** and **P II**) or at the start of 24 h preoxidization of catecholamine (protocol **P III**).

Firstly, the cytotoxic effects of catecholamines 6-OHDA and DA (1–300 μM—freshly prepared or 24 h-preincubated) were assayed (Supplementary Fig. S1). Each of them showed dose-dependency of cytotoxic effect, where all proved to be sufficiently effective at 100 μM concentration that was chosen for cytoprotection studies.

### Lactate dehydrogenase release assay for assessment of cytotoxicity

Cellular viability was determined using the assay based on measurement of lactate dehydrogenase (LDH) activity as an index of membrane damage^38–40^. Differentiated PC12 cells seeded in 96-well plates at a density of 30,000 cells per cm^2^ were incubated with compounds under investigation (alone or in combinations) for indicated time period. To determine the total cellular LDH level, positive control cells were treated with lysis buffer (0.1 M potassium phosphate, 1% Triton X-100, 1 mM DTT, 2 mM EDTA, pH 7.8, 15 min at room temperature).

A sample of the culture medium was taken from each experimental well, and the activity of LDH was assayed in Tris–HCl buffer (pH 8.9) containing 35 mM lactic acid and 5 mM NAD^+^. The rate of NAD^+^ reduction was monitored spectrophotometrically at λ = 340 nm for 2 min at RT using a microplate spectrophotometer Tecan Infinite 200 M (Tecan, Austria). The slope of the linear region was calculated, and the data were expressed as a percent of total LDH in completely lysed control (100%).

### Sytox Green nucleic acid stain for assessment of cytotoxicity

To confirm the cellular viability, another cytotoxicity assay was used. SYTOX Green nucleic acid stain, at the final concentration of 3 µM (Invitrogen-Molecular Probes, U.S.A.). Differentiated PC12 cells, seeded in 96-well plates at the same density as for LDH, were incubated with compounds under investigation using **PI** protocol or alone for up to 72 h. The fluorescence was measured at 0, 24, 48 and 72 h to observe the toxicity development over time. To determine the total ammount of cells per well, they were treated with lysis buffer at the end of the experiment (8% Triton X-100 for one hour, 37 °C). The percentage of living cells were compared to the untreated control cells (DMSO).

SYTOX fluorescent dye does not cross intact membranes but penetrates compromised membranes. Upon binding nucleic acids, it exhibits > 500-fold fluorescence enhancement. The fluorescent was measured at λ = 490 nm excitation and λ = 520 nm emission wavelengths using Tecan Infinite 200 M micro-plate spectrophotometer (Tecan, Austria).

### Epifluorescence microscopy for assessment of cellular morphology and apoptosis/necrosis induction

Cells were observed using an inverted epifluorescence microscope Nikon Eclipse TS100 with 10–40 × air objectives (Nikon, Japan) equipped with a digital camera 1300Q (VDS Vosskühler, Germany) and the software NIS-Elements AR 3.10 (Laboratory Imaging, Czech Republic). The cellular viability was visualized using nuclei staining with Hoechst 33,342 (Molecular Probes/Invitrogen, U.S.A.) and propidium iodide (Molecular Probes). Hoechst 33,342 is a blue-fluorescent probe (λ_ex_ = 360 nm, λ_em_ = 460 nm) staining all nuclei. In apoptotic cells, chromatin condensation occurs, and apoptotic cells can be identified as those with condensed and more intensely stained chromatin. The red-fluorescent (λ_ex_ = 560 nm, λ_em_ = 630 nm) DNA-binding dye, propidium iodide, cannot cross the plasma membrane of living cells, but readily enters necrotic (or late-stage apoptotic) cells and stains their nuclei red. PC12 cells seeded in 96-well plates at a density of 30,000 cells per cm^2^ were incubated with compounds under investigation (alone or in combinations) for 24 h. After that, cells had been stained for 10 min at 37 °C with 10 μg/mL Hoechst 33,342 and 1 μg/mL propidium iodide, twice washed with PBS and then assayed on the microscope.

### 2′,7′-Dichlorodihydrofluorescein diacetate assay for determination of cellular reactive oxygen species formation

To assess cellular ROS generation, the measurement of 2′,7′-dichlorodihydrofluorescein diacetate (H_2_DCF-DA; Molecular Probes) fluorescence intensity was used. This originally non-fluorescent reagent diffuses passively through the cellular membrane into the cell, where acetate groups are cleaved by intracellular esterases and subsequently oxidized by ROS formed within the cell (particularly by hydroxyl radicals; •OH) to green-fluorescent 2′,7′-dichlorofluorescein (DCF; λ_ex_ = 485 nm, λ_em_ = 525 nm). The fluorescence intensity is generally proportional to the •OH concentration, although several organic radicals (including thiyl radicals), as well as cytochrome c, may also be determined by this assay^41^.

PC12 cells seeded in a 96-well plate at a density of 30,000 cells per cm^2^ were washed with ADS buffer (116 mM NaCl, 5.3 mM KCl, 1.2 mM MgSO_4_, 1.13 mM NaH_2_PO_4_, 5 mM glucose, 1 mM CaCl_2_, 20 mM HEPES; pH 7.4) and loaded with 10 μM H_2_DCF-DA. After 60 min incubation at 37 °C, the loading buffer was discarded, and the cells were washed with ADS buffer and exposed to compounds under investigation (alone or in combinations). Fluorescence intensity was measured for 30 min at 37 °C using a microplate spectrophotometer Tecan Infinite 200 M. ROS formation in the experimental groups was expressed as a percentage of the untreated control (100%).

### Data analysis

The statistical software SigmaStat for Windows 3.5 (Systat Software, CA, U.S.A.) and GraphPad Prism version 9 (GraphPad Software, San Diego USA) were used in this study. The Grubbs test was used for detection of outlier values. For comparisons of two groups, either Student’s t-test or the nonparametric Mann–Whitney rank sum test was used. For multiple comparisons, either one-way ANOVA with Bonferroni post hoc analysis or one-way ANOVA on ranks with Dunn's post hoc analysis (data without normal distribution) were used. Differences between groups were considered to be statistically significant at a significance level p ≤ 0.05. The concentrations of compounds under investigation inducing 50% protection of cellular viability from toxicity induced by catecholamines (EC_50_ values) were calculated with CalcuSyn 2.0 software (Biosoft, U.K.).

## Results

### Comparison of various Fe chelators for protection against the toxicity of 6-OHDA

**Figure 3:**
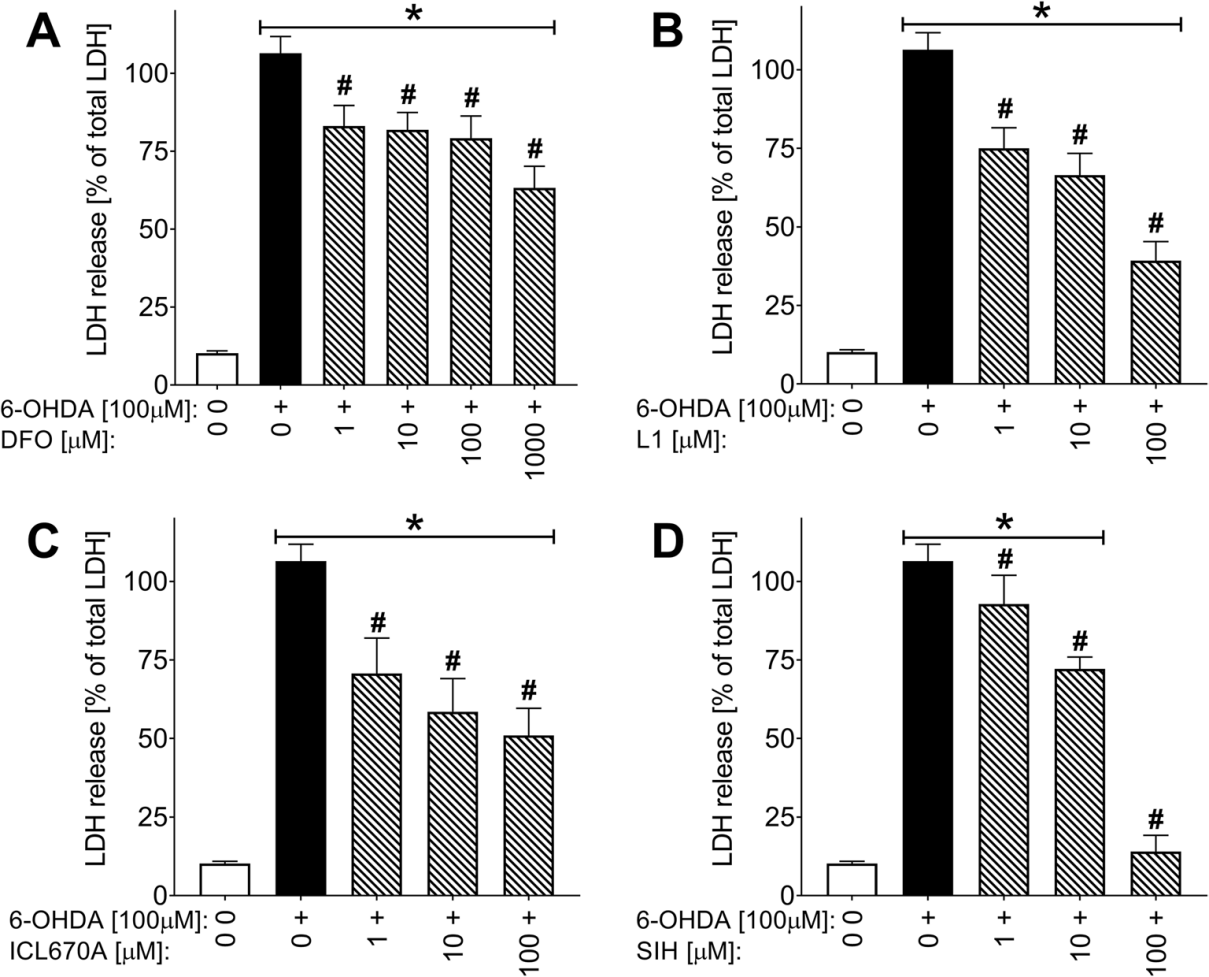
Comparison of protective effects of Fe chelators DFO, ICL670A, L1 and SIH against 6-hydroxydopamine (6-OHDA)-induced toxicity in differentiated PC12 cells. Cellular toxicities were determined using LDH activity assay and expressed as a percentage of the LDH released from completely lysed control cells. Studied Fe chelators were added to freshly-prepared 6-OHDA in the medium before the start of 24 h cellular experiments (protocol **PI**). Data are presented as means ± *SD*; *n* ≥ 4; Statistical significance (ANOVA, *p* ≤ 0.05): *vs. control group, ^#^*vs.* 6-OHDA group.

In the first set of experiments, the experimental Fe chelator SIH was compared with clinically used chelators DFO, L1, and ICL670A. For the initial comparison of the neuroprotective effects of all assayed chelators against the catecholamine toxicity, the differentiated PC12 cells were incubated for 24 h simultaneously with freshly prepared 6-OHDA (100 µM) and various concentrations of chelators (Protocol P I; Fig. 2). Cytotoxicity was determined using the LDH release assay. All studied chelators significantly protected the cells in a dose-dependent manner (Fig. 3). However, DFO was not able to reduce the toxicity of 6-OHDA by 50%, even in the highest tested concentration (1000 µM). L1, ICL670A, and SIH showed rather similar protective effects, and their EC_50_ values were 23.4 ± 9.9 µM, 21.6 ± 10.9 µM, and 20.3 ± 9.7 µM, respectively. At 100 µM (the highest concentration at which all the lipophilic chelators could be dissolved), SIH achieved the best efficiency as it reduced the toxicity of 6-OHDA so that the cellular viability did not differ significantly from the untreated control cells.

### Comparison of toxicities of Fe chelators

**Figure 4:**
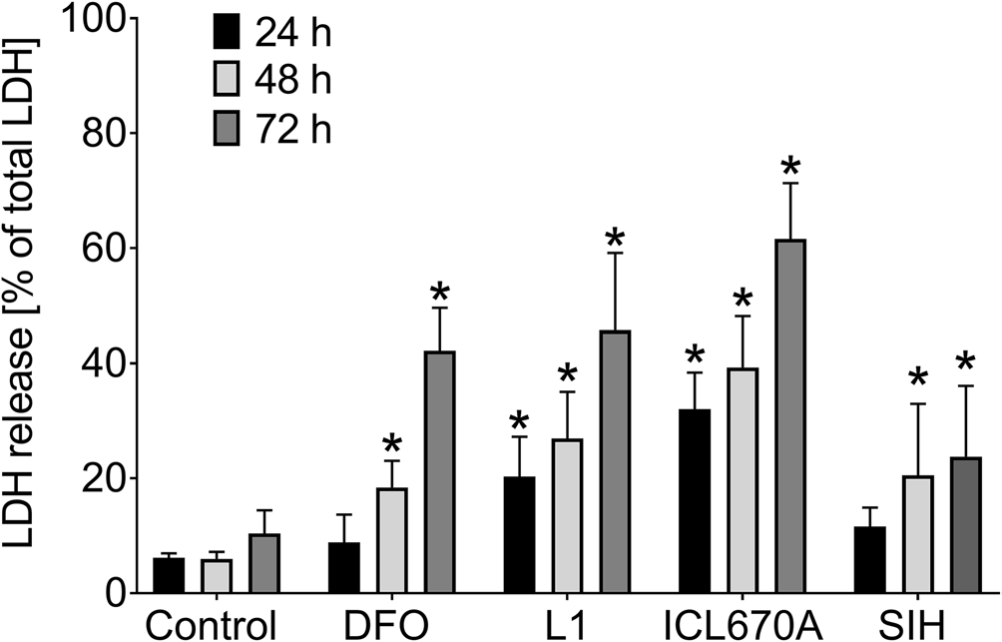
Comparison of own cytotoxic effect of Fe chelators DFO, ICL670A, L1 and SIH (all 100 µM) in differentiated PC12 cells. Cellular toxicities were determined after 24 h, 48 h or 72 h incubation using LDH activity assay and expressed as a percentage of the LDH released from completely lysed control cells. Data are presented as means ± *SD*; *n* ≥ 4; Statistical significance (ANOVA, *p* ≤ 0.05): *vs. control group.

Inherent cytotoxicities of studied Fe chelators (all in 100 µM concentration, at which the chelators were protective) were assessed by their incubations for 24 h, 48 h, or 72 h with differentiated PC12 cells. As seen in Fig. 4, SIH showed the lowest cytotoxicity, which was particularly evident in longer cellular exposures (48 h and 72 h), although the cytotoxicity was still statistically significant compared to control. DFO displayed generally similar results as SIH, although it was more toxic than SIH in the longest incubation (72 h). Orally active chelators L1 and ICL670A showed a statistically significant increase in cytotoxicity even after 24 h incubation. ICL670A displayed the highest cytotoxicity. Using SYTOX fluorescence measurements, practically no toxicity of all tested compounds was observed at 24 h compared to control (DMSO). As with LDH, longer exposures (48 and 72 h) resulted to higher toxicity, mostly in ICL670A. SIH displayed lower toxicity after 24 h of incubation; nevertheless, BSIH was not toxic for up to 72 h, where it displayed the same viability as untreated control cells (Fig. 8).

### Comparison of the cytoprotective potential of chelator SIH and prochelator BSIH

The comparison of diverse Fe chelators revealed that although some protection can be a general property of Fe chelators, the lipophilic agents (L1, ICL-670A, SIH) have higher potential to exert neuroprotection, apparently due to the better access to intracellular labile Fe pool. However, an important issue is the own toxicity of chelators, as in states without Fe overload (such as in PD), Fe chelation can induce severe toxicity due to the depleting or withholding of this important biogenic metal. To this end, the concept of prochelators, which release active chelating agents only upon oxidative injury, has been introduced.

Hence, the Fe chelator SIH that displayed the most favorable properties with respect to both efficiency and own toxicity was studied in more detail together with its prochelator derivative BSIH. In this pivotal set of experiments, the protective effects of SIH and BSIH were assayed in a wide concentration range (3–600 μM) in cells that were challenged by two catecholamines, 6-OHDA, and DA (both 100 μM). T Three different incubation protocols have been used to dissect the role of auto-oxidation in catecholamine neurotoxicity and the potential of SIH and BSIH to prevent it (Fig. 2).

**Figure 5:**
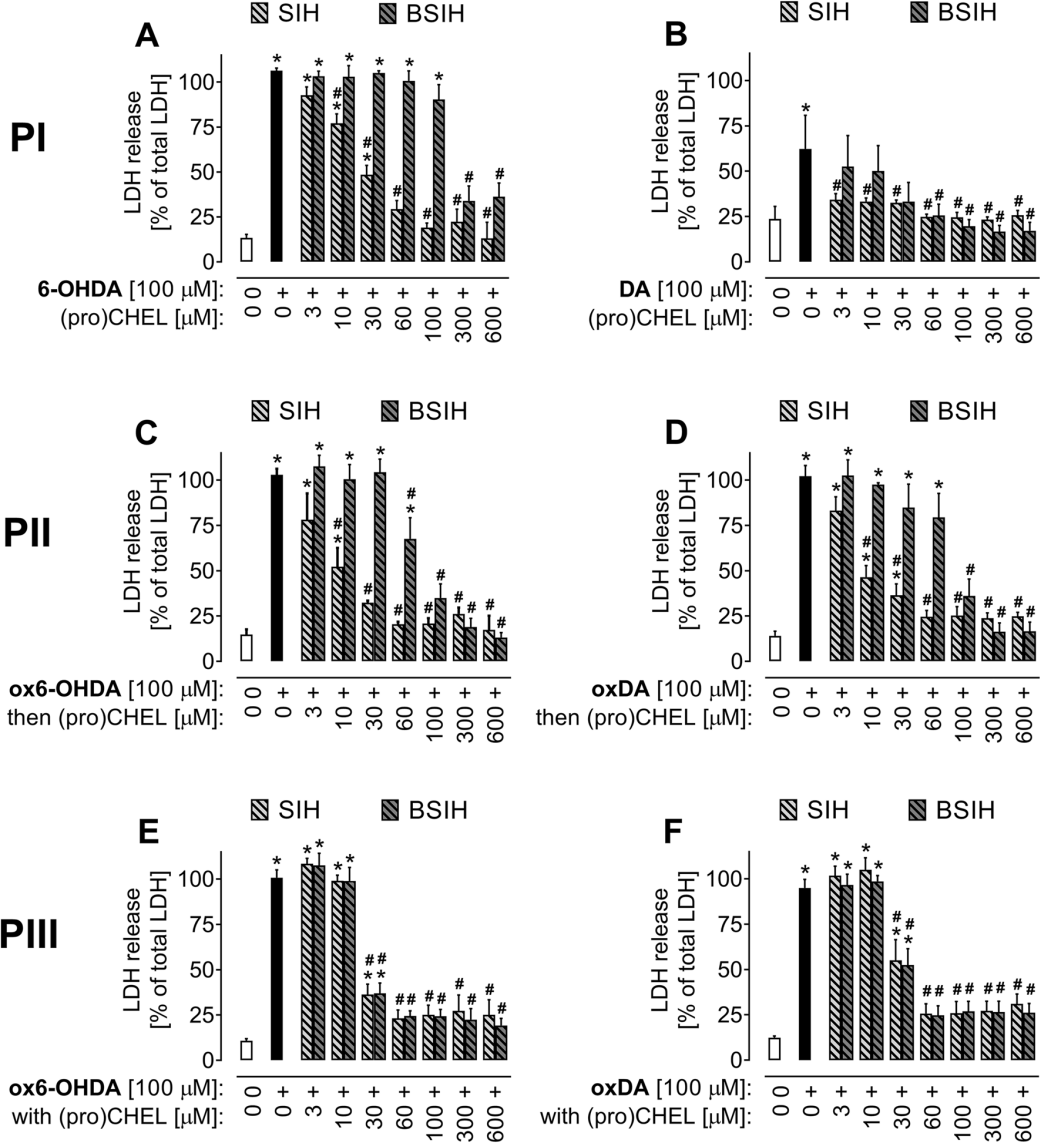
Comparison of protective effects of the Fe chelator SIH and prochelator BSIH against the toxicity of 6-hydroxydopamine (6-OHDA)- and dopamine (DA) in differentiated PC12 cells. Cellular toxicities were determined using LDH activity assay and expressed as a percentage of the LDH released from completely lysed control cells. (**A, B**) SIH or BSIH were added to freshly-prepared catecholamine, (**A**) 6-OHDA or (**B**) DA before the start of 24 h cellular experiments (protocol **PI**); (**C, D**) SIH or BSIH were added immediately before cellular experiments to 24 h-preoxidized catecholamines, (**C**) ox6-OHDA or (**D**) oxDA (protocol **PII**); (**E, F**) SIH or BSIH were preincubated for 24 h together with catecholamines, (**E**) ox6-OHDA or (**F**) oxDA and then added to cells (protocol **PIII**). Data are presented as means ± *SD*; *n* = 4–5; Statistical significance (ANOVA, *p* ≤ 0.05): *vs. control group, ^#^*vs.* corresponding catecholamine group.

Firstly, we used the experimental setup where the compound under investigation was co-incubated for 24 h with freshly-prepared 6-OHDA or DA (protocol **P I,** used for the initial comparison of various chelators). As seen in Figs. 5A,B, S3, both SIH and BSIH partially protected the cells in a concentration-dependent manner. Better protection was obtained with SIH, which significantly reduced the cytotoxic effects of catecholamines already at relatively low concentrations (≥ 10 μM or ≥ 3 μM in the case of 6-OHDA or DA, respectively). BSIH exerted protection only at concentrations one order of magnitude higher than SIH, apparently due to the need for activation and delayed onset of its protective action. To expand the knowledge of the tested compounds' behavior, using the protocol PI, the investigation time was prolonged to 72 h and evaluated by SYTOX (Fig. 9A,B, Supplementary Fig S5). In SIH, co-exposed to 6-OHDA, after 24 h, comparable results were observed as for LDH and all concentrations of SIH were able to significantly protect the PC12 cells. At 48 and 72 h incubations, all the concentrations were able to significantly protect the cells as well, but with a decreasing efficiency (Fig. 9A). With BSIH, its concentrations 100 to 600 µM were able to partially, but significantly protect the cells at all the times.

Then the protective potentials of SIH and BSIH against the toxicities of catecholamines were assayed in setup, when the cells were incubated with studied (pro-)chelator added immediately before cellular experiments to medium with 24 h-preincubated (i.e., oxidized) catecholamines—ox6-OHDA or oxDA (Fig. 5C,D; protocol **P II**). As seen in Fig. 5C,D, both SIH and BSIH exerted protection in a dose-dependent manner. However, whereas at lower concentrations (≤ 100 µM), SIH was considerably more efficient than BSIH, at concentrations ≥ 300 µM BSIH was able to reduce the toxicities of ox6-OHDA and oxDA slightly better than SIH. Finally, in a setup when cells were incubated with studied compounds added to the medium at the beginning of catecholamine 24 h-preoxidation (“co-preincubation”, protocol **P III**), both SIH and BSIH were able to significantly reduce cytotoxicity induced by ox6-OHDA and oxDA, albeit only at concentrations ≥ 30 µM (Fig. 5E,F). A summary of the calculated EC_50_ values is shown in Table 1.

**Table 1:** Comparison of protective effects of Fe chelator SIH and its derived prochelator BSIH against toxicities of catecholamines 6-hydroxydopamine (6-OHDA) and dopamine (DA) and 24 h-preincubated catecholamines (ox6-OHDA, oxDA) using various experimental protocols and LDH assay.

	(Pro) chelator	Protocol P I		Protocol P II		Protocol P III	
		6-OHDA	DA	ox6-OHDA	oxDA	ox6-OHDA	oxDA
EC_50_ [µM]	SIH	20 ± 10	< 3	4 ± 2	7 ± 4	21 ± 5	30 ± 13
	BSIH	174 ± 64	25 ± 6	65 ± 22	90 ± 30	21 ± 4	29 ± 8

**Protocol P I**—the (pro)chelator was co-incubated for 24 h with freshly-prepared catecholamine; **Protocol P II**—the cells were incubated with studied (pro)chelator added just before cellular experiments to medium with 24 h-preincubated (i.e., oxidized) catecholamines; **Protocol P III**: cells were incubated with studied compounds added to the medium at the beginning of catecholamine 24 h-preoxidation. EC_50_ values [µM] are calculated concentrations of (pro) chelator at which it reduces the toxicity of catecholamine to 50%.

Furthermore, these results were confirmed by epifluorescence microscopy with nuclear co-staining by fluorescent probes Hoechst 33342 and propidium iodide. As seen in Supplementary Fig. S4, epifluorescence images reflect the pronounced toxicity of 100 µM 6-OHDA (induction of necrosis and/or late-stage apoptosis) and the ability of SIH and BSIH to protect cellular viability against apoptosis/necrosis induced by 6-OHDA. Whereas both SIH and BSIH (100 µM) were similarly protective in protocols II and III (with 24 h catecholamine preoxidation), in protocol I, using fresh 6-OHDA, SIH induced more pronounced protection than BSIH at this concentration (100 µM), which is in agreement with the LDH release assay (Figs. 5A, 9).

### Comparison of cytotoxicity of SIH and BSIH

**Figure 6:**
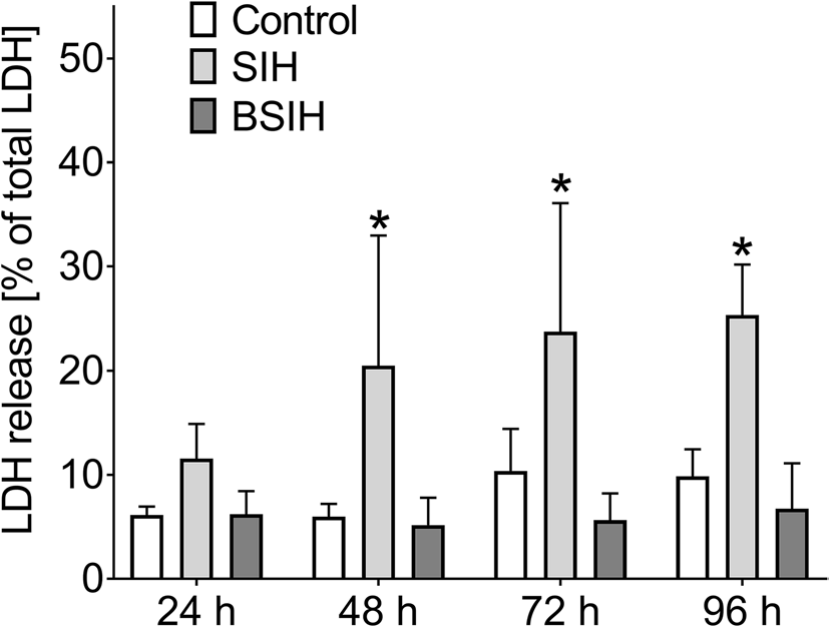
Comparison of cytotoxic effects of Fe chelator SIH and prochelator BSIH in differentiated PC12 cells. Cellular toxicities were determined using LDH activity assay and expressed as a percentage of the LDH released from completely lysed control cells. Time-dependency of cytotoxic effects of SIH and BSIH, both 100 µM, following incubations for up to 96 h with cells. Data are presented as means ± *SD*; *n* = 4; Statistical significance (ANOVA, *p* ≤ 0.05): *vs. control group.

Inherent cytotoxicities of the chelator SIH and its derived prochelator BSIH were firstly determined following their 24 h incubations with differentiated PC12 cells in a concentration range from 3 to 600 µM (solubility limit). As shown in Fig. S2, SIH induced a statistically significant although rather mild increase in LDH release, whereas BSIH did not. Therefore, their toxicities were then observed in time, when both studied compounds at concentration 100 µM were incubated with cells for up to 96 h. Here, SIH induced statistically significant viability reduction during the whole experiment, whereas BSIH did not show even minor signs of cytotoxicity. Moreover, BSIH exerted even certain protection of differentiated PC12 cells against spontaneous cell death during prolonged experiments (Fig. 6). With SYTOX measurements, comparable results were obtained, with notable no significant difference between control and BSIH (Fig. 8).

Low toxicities of SIH and BSIH after 24 h incubation are also apparent from epifluorescence microscopy images of nuclei stained with Hoechst 33,342 and propidium iodide. As seen in Supplementary Fig. S4, the proportion of cells with apoptotic/necrotic features was comparable to that of untreated control.

### Determination of intracellular reactive oxygen species formation

The H_2_DCF-DA assay was used to assess the ability of SIH and BSIH to prevent the generation of intracellular ROS, measured as fluorescence intensity at the end of 30-min incubation of cells with SIH or BSIH (100 µM) with freshly-prepared 6-OHDA (protocol **P I**) or 24 h-preoxidized ox6-OHDA (protocols **P II** and **P III**).

**Figure 7:**
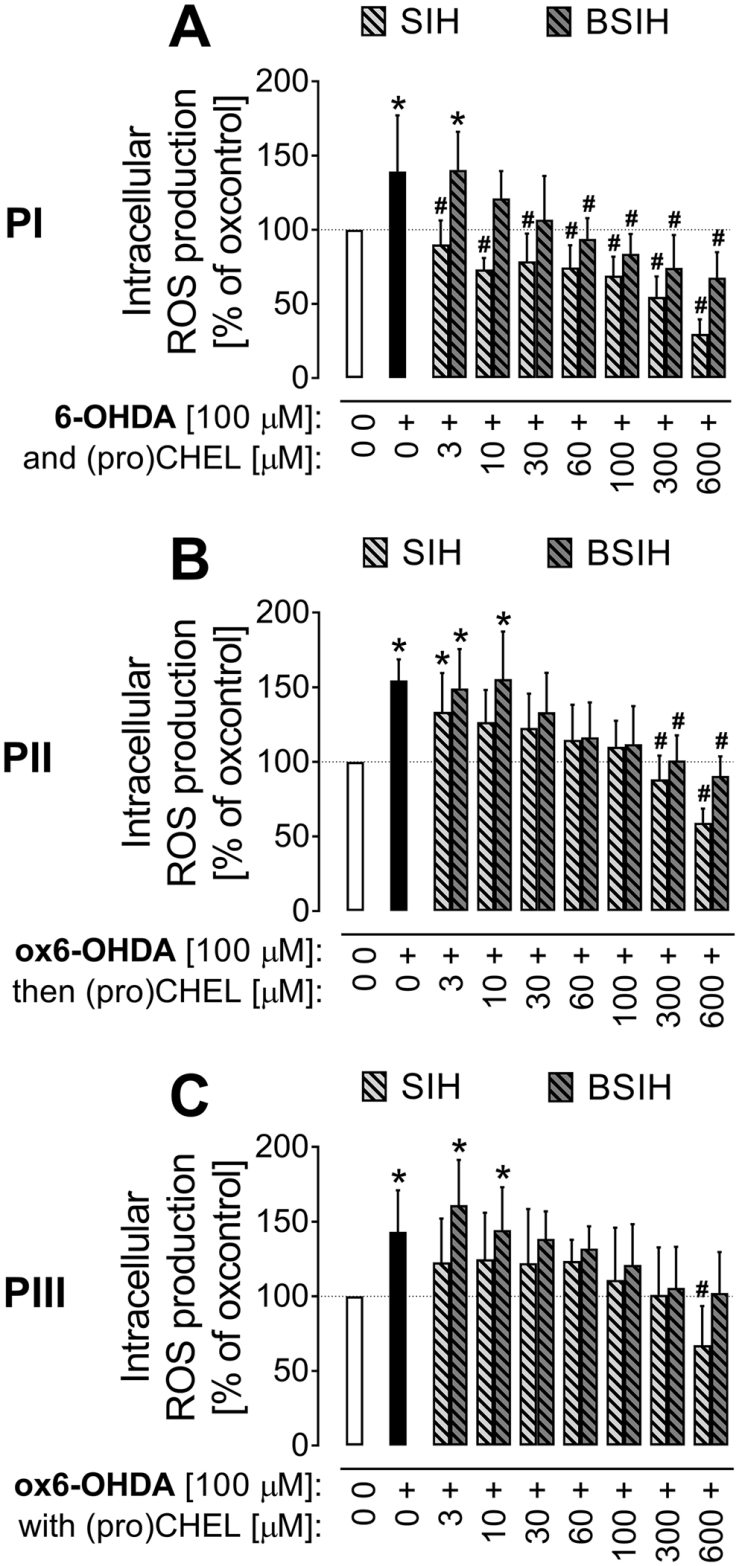
Comparison of effects of Fe chelator SIH and prochelator BSIH on cellular oxidative stress induced by 6-hydroxydopamine (6-OHDA) and its 24 h-preoxidized form (ox6-OHDA). Intracellular reactive oxygen species (ROS) formation was determined by H_2_DCF-DA assay following the 30 min treatment of differentiated PC12 cells with a combination of 6-OHDA (100 µM) and Fe (pro)chelator (0–600 µM). ROS production (intracellular fluorescence) was expressed as a percentage of the untreated control group. Cells were incubated for 24 h with various concentrations of SIH or BSIH added to: freshly-prepared 6-OHDA (**A**; protocol **PI**); to medium with 24 h-preoxidized 6-OHDA (**B**; protocol **PII**); or to medium with 6-OHDA at the beginning of 24 h preincubation (**C**; protocol **PIII**). Data are presented as means ± *SD*; *n* = 4; Statistical significance (ANOVA, *p* ≤ 0.05): *vs. control group, ^#^*vs.* corresponding catecholamine group.

6-OHDA and ox6-OHDA (both 100 µM) induced a statistically significant increase in oxidation of H_2_DCF to fluorescent DCF. Co-incubation with both SIH and BSIH significantly decreased ROS formation (Fig. 7), with SIH being in most experiments more effective than BSIH. The most effective protection was observed in the setup with fresh 6-OHDA and SIH/BSIH (Fig. 7A).

## Discussion

Disturbance of brain Fe homeostasis has been implicated in neuronal injury and death in various neurodegenerative diseases, such as PD, Alzheimer's disease, Friedreich's ataxia, or amyotrophic lateral sclerosis. Oxidative stress may lead to neuronal cell damage and/or loss due to the diverse processes that (depending on the mode and/or intensity of the stimulus) include necrosis, apoptosis, autophagy or ferroptosis, a relatively novel type of programmed cell death dependent on Fe and characterized by the accumulation of various lipid peroxides^42^. Furthermore, Fe may contribute to neurodegenerative changes by various other mechanisms than only by production of ROS—e.g*.*, Fe may enhance the translation of α-synuclein mRNA level^43^ or the imbalance in Fe homeostasis affects the expression of DA D2 receptors^44^.

Fe chelators have been suggested as neuroprotective and neurorestorative in various neurological diseases, suggesting that Fe chelation might be a promising treatment modality^45,46^. Apart from preventing Fe from entering the Haber–Weiss reaction and subsequent reduction of ROS formation, Fe chelators can induce HIF-1alpha that modulates the expression of several key genes involved in neuroprotection^47^.

Previously, several studies evaluated the potential of Fe chelators for neuroprotection. Focus has been particularly on registered agents (mostly used in clinics to manage Fe accumulation in transfusion-dependent anemias) with known pharmacokinetic and safety profiles. DFO has been the first Fe chelator used in clinical practice to manage diseases associated with chronic Fe overload and Chouraqui et al*.* showed that DFO (10–30 μM) reduced the toxicity induced by glutathione depletion agent buthionine sulfoximine in PC12 cells^48^. DFO also significantly attenuated methamphetamine- or 6-OHDA-induced neurotoxicity in rats^49,50^. However, DFO is a hydrophilic Fe chelator with a rather high molecular weight that does not readily pass through biological membranes. Orally active chelators are deferiprone (L1) and deferasirox (ICL-670A). Molina-Holgado et al*.* have shown in primary mouse cortical neurons significant protective action of L1 against various insults, including Fe^3+^ (ferric nitrilotriacetate), H_2_O_2_ and aggregated forms of amyloid-beta^14^. SIH, although studied in various cells, isolated organs or in in vivo experiments, has very limited data available on protection of neurons or derived cells. It has been studied by Shi et al*.* and significantly reduced the toxicity of 6-OHDA in SH-SY5Y cells from ≈ 60 to ≈ 40%, although the SIH treatment itself also led to ≈ 40% decrease in cell viability^51^.

The present study is the first that directly compares all three Fe chelators used in clinical practice with SIH and its prochelator BSIH. PC12 cells were used as they have the capacity to undergo neuronal differentiation in response to NGF. Using this approach, the cell population was before the experiments turned to a postmitotic phenotype with many features of sympathetic and dopaminergic neurons. Apart from DA (a natural neurotransmitter of the brain), 6-OHDA (also known as oxidopamine) was used as it is a well-established and long-used model neurotoxic agent. The local injection of 6-OHDA into the midbrain of rats and mice causes an acute degeneration of dopaminergic neurons^52^. 6-OHDA is recognized by nigral neurons as DA and is taken up by the cells. With its entrance into the cytoplasm, 6-OHDA expresses its toxicity and destroys monoaminergic cells.

**Figure 8:**
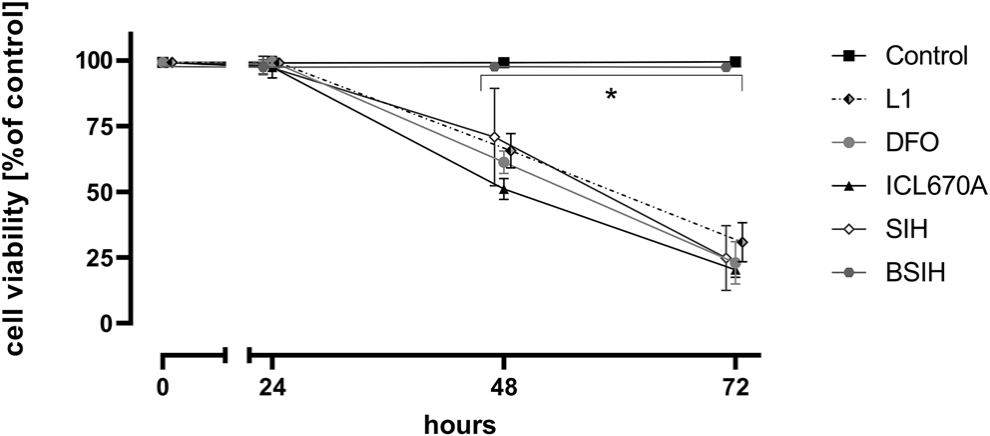
Comparison of own cytotoxic effect of Fe chelators DFO, ICL670A, L1, SIH and prochelator BSIH (all 100 µM) in differentiated PC12 cells. Cellular toxicities were determined after 24 h, 48 h and 72 h incubation using SYTOX measurement, expressed as a percentage of viable cells and were related to untreated control (DMSO). Data are presented as means ± *SD*; *n* ≥ 4; Statistical significance (ANOVA, *p* ≤ 0.05): *vs. Control group.

For the initial comparison of neuroprotective effects of studied chelators of Fe (DFO, L1, ICL670A and SIH), freshly prepared 6-OHDA was used in 100 µM concentration. This concentration correlated with other studies^20,53^ and we also verified that this concentration induced robust viability loss of PC12 cells (Fig. S1A) giving ample room for assessing potential cytoprotective agents. All the assayed chelators showed a dose-dependent decrease of a neurotoxic effect of 6-OHDA (Fig. 3). SIH displayed the best neuroprotective properties, especially in 100 µM concentration. SIH also showed the lowest inherent toxicity (Figs. 4, 8) and, therefore, the most favorable ratio of own toxicity and cytoprotective efficiency. These findings confirmed the results of previous studies, where SIH showed advantageous properties in comparison with all three clinically used Fe chelators on H9c2 cardiomyocyte-derived cells against cellular oxidative damage induced by hydrogen peroxide, *tert*-butylhydroperoxide or catecholamines—epinephrine and isoprenaline^22,54,55^.

**Figure 9:**
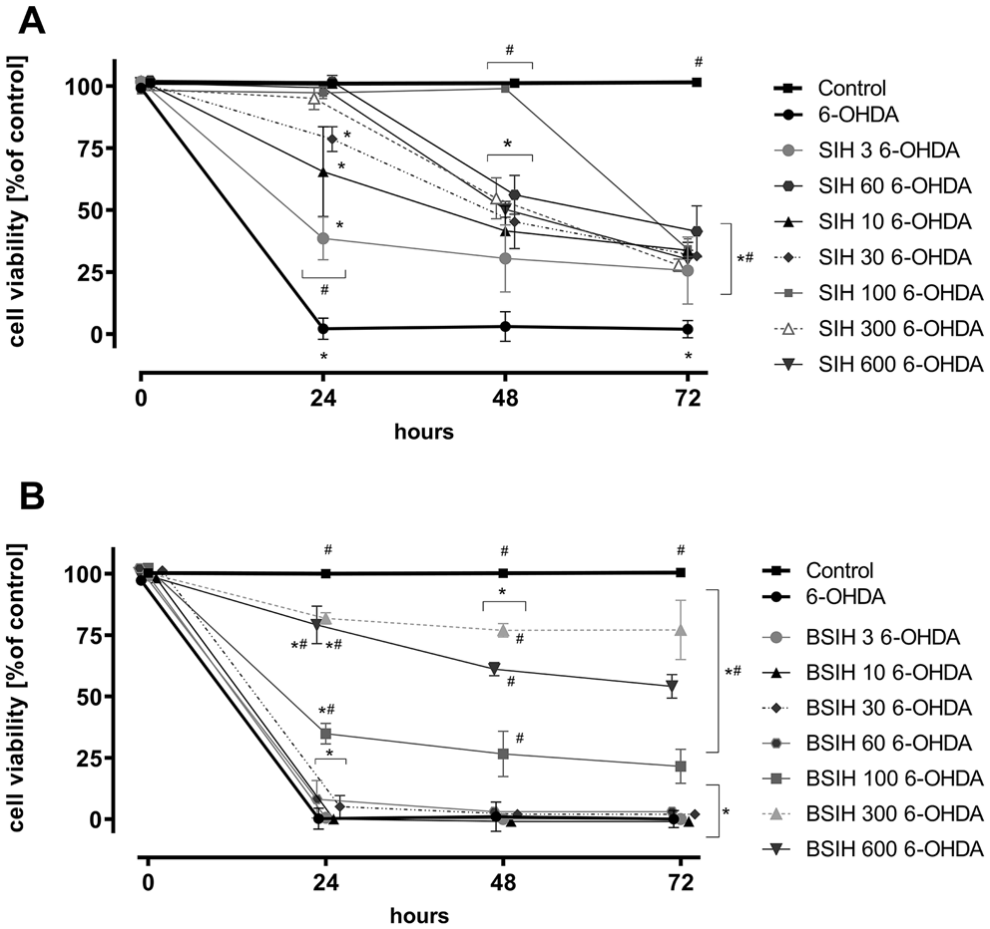
Comparison of protective effects of the Fe chelator SIH and prochelator BSIH against the toxicity of 6-hydroxydopamine (6-OHDA) in differentiated PC12 cells. Cellular toxicities were determined using SYTOX measurement and expressed as a percentage of viable cells and were related to untreated control. SIH (**A**) or BSIH (**B**) were added to the cells with freshly-prepared 6-OHDA (protocol **PI**). Data are presented as means ± *SD*; *n* ≥ 4; Statistical significance (ANOVA, *p* ≤ 0.05): *vs. control group, ^#^*vs.* 6-OHDA. Statistical significance is also displayed in tables in Supplementary Figure S5.

In the second part of this study, we focused on a more detailed examination of SIH and BSIH. The strategy of oxidative-stress activated prochelators in principle allows targeted chelation therapy for pathological states not connected with systemic Fe overload but rather with local Fe disbalance contributing to cellular injury. Their cytoprotective properties were assessed against toxicity caused by freshly prepared or 24 h-preincubated 6-OHDA and DA (Figs. 5, 9, S3). DA is a natural neurotransmitter of the brain whose toxicity closely resembles those of 6-OHDA and may involve both extracellular and intracellular generation of ROS by oxidation of the catechol moiety^20^. SIH, as well as BSIH, showed significant and dose-dependent cytoprotective effects against toxicity induced by both 6-OHDA and DA. Here, SIH displayed better efficacy in lower concentrations than BSIH (Figs. 5A–D, 9, S3; Table 1—**PI-PII**), probably due to the limited time to activate prochelator BSIH to active chelating products. This hypothesis was confirmed by subsequent experiments, where SIH or BSIH was preincubated together with 6-OHDA or DA for 24 h and where BSIH showed essentially the same cytoprotective effect as SIH (Fig. 5E,F; Table 1—**PIII**). Both compounds, SIH and BSIH, displayed very low inherent toxicity following 24 h incubation (Figs. 6, 8, Fig. S2). BSIH (100 µM) did not show any inherent toxicity even after 96 h exposure to differentiated PC12 cells. These findings correlated with a previous study, where BSIH (600 µM; the highest achievable concentration) did not induce any significant viability loss on H9c2 cells even after 168 h cellular exposure^54^. The low inherent toxicity of this compound, as well as its promising cytoprotective effect, was verified using epifluorescence microscopy (Fig. S4).

**Figure 10:**
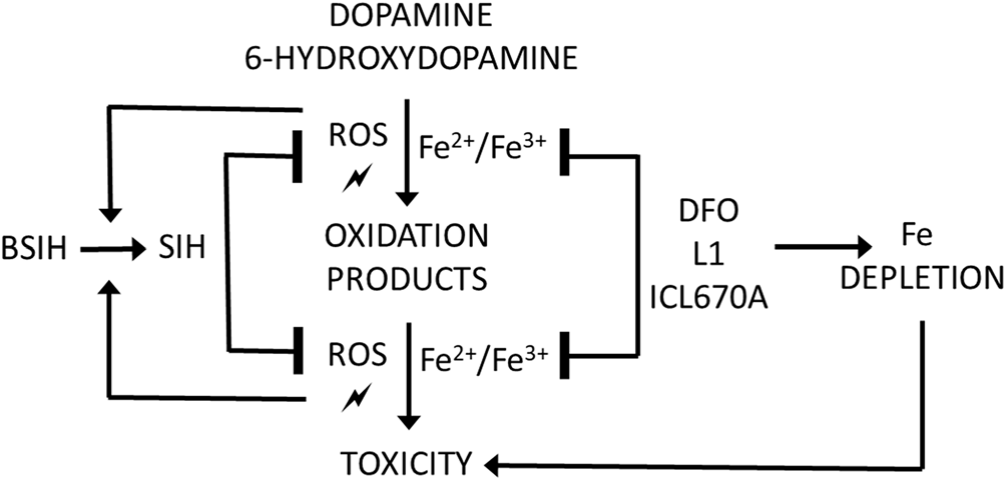
Proposed working model of iron (pro)chelators protecting the PC12 cells against 6-hydroxydopamine- and dopamine-induced oxidative stress and toxicity.

In conclusion, the present study confirmed that chelation of free redox-active cellular Fe may be beneficial in the mitigation of catecholamine neurotoxicity. We observed advantageous properties of experimental Fe chelator SIH and its prochelator BSIH. Both compounds showed the ability to significantly reduce intracellular ROS generation and further oxidative stress propagation, resulting in a promising cytoprotective effect. Although BSIH required higher concentrations than SIH to achieve the same protective effect, its greatest advantage is the absence of its inherent toxicity (Fig. 10).

The present work is an initial in vitro toxicological study using rather high catecholamine concentrations and its relevance to human neurological diseases needs to be assessed using more complex in vivo models of neurodegenerative diseases with pathophysiologically-relevant catecholamine levels. Future studies should also focus on the pharmacokinetic aspects of the (pro)chelators, including metabolization, excretion, eventual accumulation in tissues and, most importantly, their ability to cross the blood–brain barrier, which is an important property of all compounds intended for the treatment of neurodegenerative diseases.

## Supplementary Information

Supplementary Information.
