# Supplementary material for: Examination of diverse iron-chelating agents for the protection of differentiated PC12 cells against oxidative injury induced by 6-hydroxydopamine and dopamine
Source: Sci Rep. 2022 Jun 13;12:9765. doi: 10.1038/s41598-022-13554-x (PMC9192712; doi:10.1038/s41598-022-13554-x)
Supplement: Supplementary file 1 — Supplementary Information. [file 41598_2022_13554_MOESM1_ESM.docx]

**Examination of diverse iron-chelating agents for the protection of differentiated PC12 cells against oxidative injury induced by
6-hydroxydopamine and dopamine**

**Supplementary information**

**Supplementary Figure S1**


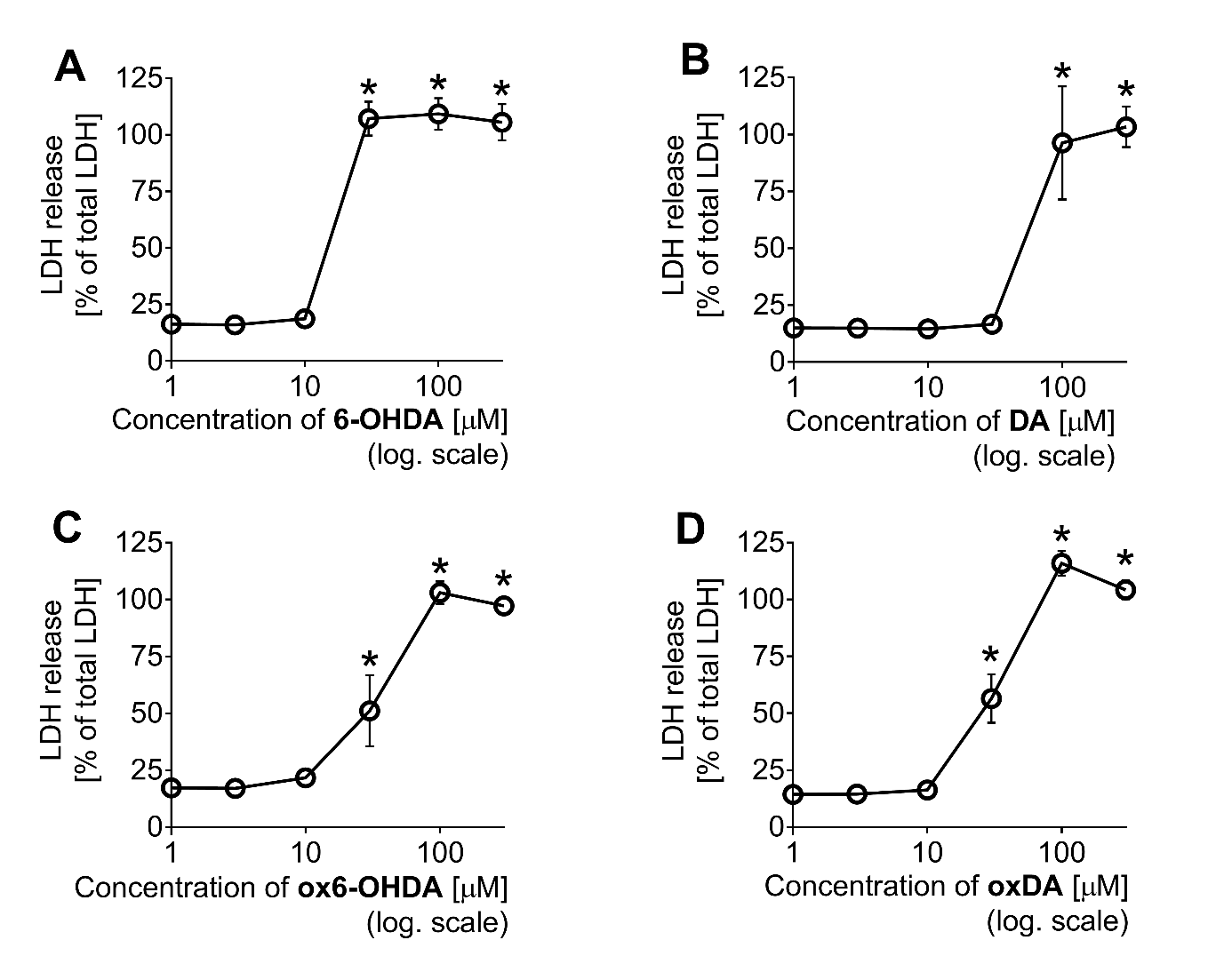


**Comparison of cytotoxic effects of studied catecholamines in differentiated PC12 cells.** Cellular viabilities were determined by LDH release assay and expressed as a percentage of the LDH released from completely lysed control cells. Own toxicities of (A) 6‑OHDA, (B) DA, (C) ox6-OHDA and (D) oxDA following 24h incubations with cells. Data are presented as means ± *SD*; *n* = 4; Statistical significance (ANOVA, *p* ≤ 0.05): * *vs.* control group.

**Supplementary Figure S2**


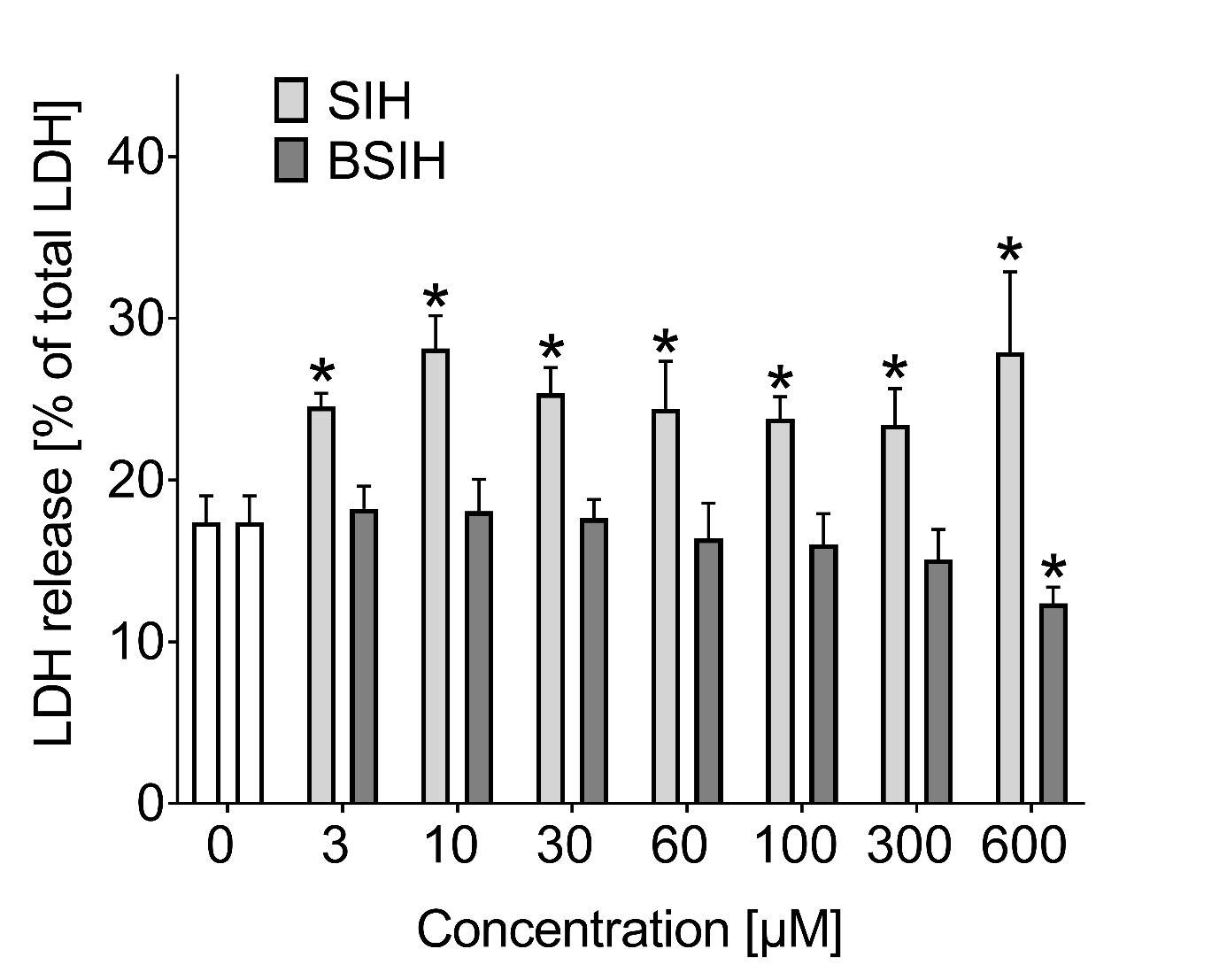


**Comparison of cytotoxic effects of Fe chelator SIH and prochelator BSIH in PC12 cell line differentiated into sympathetic neurons.** Cellular viabilities were determined using LDH release assay and expressed as a percentage of the LDH released from completely lysed control cells. Dose-dependency of cytotoxic effect of SIH and BSIH following 24h incubations with cells. Data are presented as means ± *SD*; *n* = 4; Statistical significance (ANOVA, *p* ≤ 0.05): * *vs.* control group.

**Supplementary Figure S3**


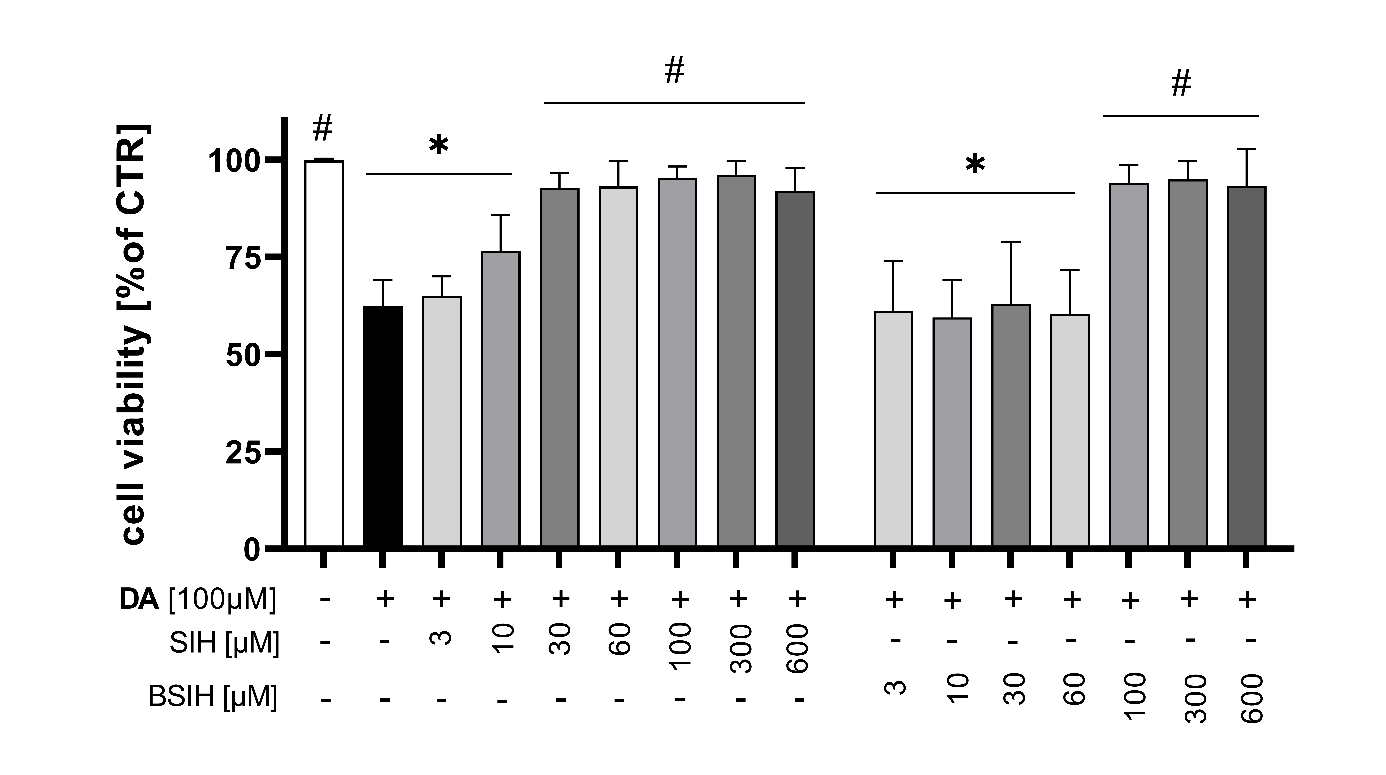


**Comparison of protective effects of the Fe chelators SIH and prochelator BSIH against the toxicity of dopamine (DA) in differentiated PC12 cells.** Cellular viabilities were determined using SYTOX measurement and expressed as a percentage of viable cells and were related to untreated control. SIH and BSIH were added together with the freshly prepared DA (protocol **PI**). Data are presented as means ± *SD*; *n* = 4; Statistical significance (ANOVA, *p* ≤ 0.05): * *vs.* control group, ^#^ vs. DA.

**Supplementary Figure S4**


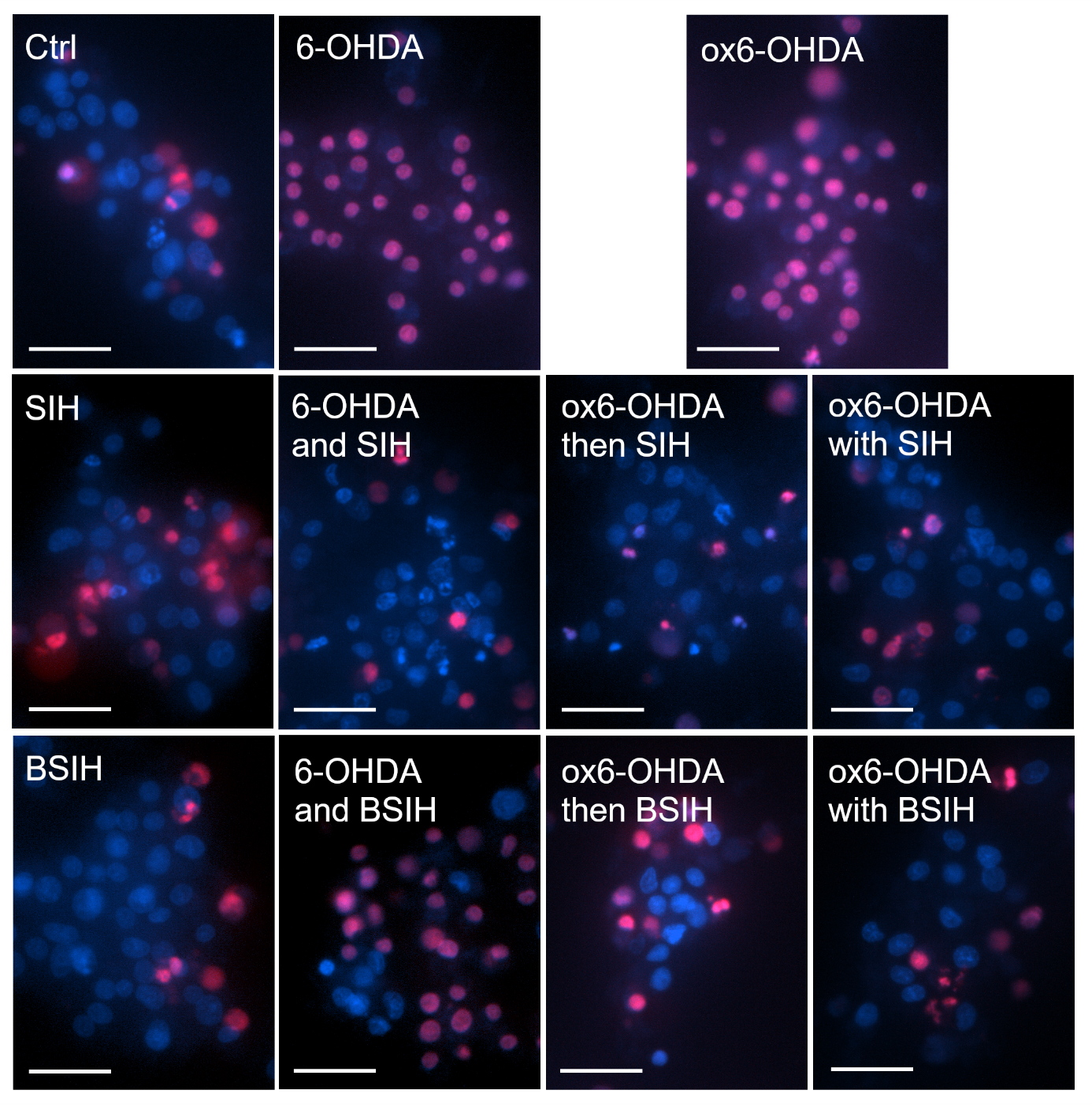


**Epifluorescence photomicrographs with nuclear stainings of differentiated PC12 cells with Hoechst 33342 and propidium iodide.** Cells were incubated for 24 h with control medium, Fe chelator SIH (100 µM), Fe prochelator BSIH (100 µM), 6‑hydroxydopamine (6-OHDA; 100 µM), 24h-preoxidized 6-hydroxydopamine (ox6-OHDA; 100 µM) alone, or in combinations, when the SIH or BSIH was added to 6-OHDA or ox6-OHDA immediately before the cellular experiment (“6-OHDA and SIH/BSIH“ – protocol **PI**; or “ox6-OHDA then SIH/BSIH“ - protocol **PII**) or at the beginning of 24h-preincubation (“ox6-OHDA with SIH/BSIH“ – protocol **PIII**). Scale bars represent 50 μm.

**Supplementary Figure S5**


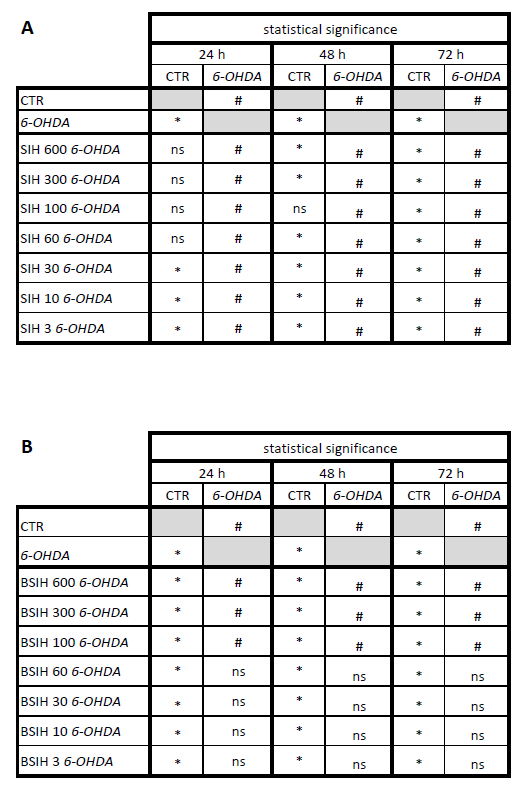


**Statistical significance of Figure 9 displayed as tables.**

**Comparison of protective effects of the Fe chelator SIH and prochelator BSIH against the toxicity of 6‑hydroxydopamine (6-OHDA) in differentiated PC12 cells.** Cellular toxicities were determined using SYTOX measurement and expressed as a percentage of viable cells and were related to untreated control. SIH (A) or BSIH (B) were added to the cells with freshly-prepared 6-OHDA (protocol **PI**). Data are presented as means ± *SD*; *n* *≥* 4; Statistical significance (ANOVA, *p* ≤ 0.05): * *vs.* control group, ^#^ *vs.* 6-OHDA.
